# CT-Based Reference Values for Splenic Artery and Vein Diameters in Individuals Aged 1–80 Years

**DOI:** 10.3390/diagnostics16142267

**Published:** 2026-07-20

**Authors:** Iklil Eryılmaz, Fırat Aslan, Serhat Binici, Uğur Yanç, Bülent Sönmez, Veysel Tahiroğlu, Burhan Beger, Muzaffer Onder Oner, Berke Bulut Yüktaşır, Barış Ten, Murat Gölpınar, Turan Koç, Orhan Beger

**Affiliations:** 1 Department of General Surgery, Kartal Dr. Lutfi Kirdar City Hospital, 34860 İstanbul, Türkiye; 2 Department of General Surgery, Faculty of Medicine, Van Yüzüncü Yıl University, 65090 Van, Türkiye; dr.aslan.2609@hotmail.com (F.A.); drserhatbinici@gmail.com (S.B.); 3 Department of Radiology, Şırnak State Hospital, 73000 Şırnak, Türkiye; yancugur@gmail.com; 4 Department of Neonatology, Van Education and Research Hospital, 65300 Van, Türkiye; sonmezbs18@gmail.com; 5 Department of Nursing, Faculty of Health Sciences, Şırnak University, 73000 Şırnak, Türkiye; veysel.tahiroglu@sirnak.edu.tr; 6 Department of Pediatric Surgery, Faculty of Medicine, Van Yüzüncü Yıl University, 65090 Van, Türkiye; burhanbeger@hotmail.com; 7 Department of General Surgery, BHT Clinic, İstanbul Nişantaşı University, 34307 İstanbul, Türkiye; zkudrooner@gmail.com; 8 Faculty of Medicine, İzmir Democracy University, 35575 İzmir, Türkiye; yuktasr@gmail.com; 9 Department of Radiology, Faculty of Medicine, Mersin University, 33079 Mersin, Türkiye; drbaristen@hotmail.com; 10 Department of Anatomy, Faculty of Medicine, Hitit University, 19040 Çorum, Türkiye; golpinarmurat@hotmail.com; 11 Department of Anatomy, Faculty of Medicine, Kahramanmaraş Sütçü İmam University, 46050 Kahramanmaraş, Türkiye; turann_koc@yahoo.com; 12 Department of Anatomy, Faculty of Medicine, Gaziantep University, 27580 Gaziantep, Türkiye; obeger@gmail.com

**Keywords:** splenic artery, splenic vein, spleen, L1, vascular disorders

## Abstract

**Objectives:** Demographic features of subjects, such as body mass index, height, weight, age, or sex, may affect diameters of vessels such as the splenic artery (SA) and splenic vein (SV). Some studies use alternative anatomical indicators, including the body of the first lumbar vertebra (L1), to improve standardization in the assessment of vascular pathologies. This study aimed to evaluate the relationship between splenic vessel diameters (SA and SV) and L1 using abdominopelvic computed tomography images obtained from individuals aged 1–80 years. **Methods:** Radiologic images of 800 subjects were included in the examination. Proximal (SA1 and SV1), middle (SA2 and SV2) and distal (SA3 and SV3) calibers of SA and SV were measured. Ratios of vessel calibers to the transverse diameter of the body of L1 (L1TD) were calculated. **Results:** Age had a clear influence on the diameters of SA and SV. SV1 showed a steady increase until the early fifties, followed by a decline beginning in the seventies. Both SV2 and SV3 enlarged until the early twenties, after which no significant variation was observed. SA1, SA2, and SA3 gradually increased from 1 year of age through the early fifties, but demonstrated a statistically significant reduction at older ages. The ratios of SA and SV diameters to L1TD were highest during the first decade of life and dropped markedly in the second decade. Following this period, these ratios generally tended to rise initially and then decline with further aging. **Conclusions:** This study provides age-specific CT-based reference values for SA and SV diameters and their ratios to the L1 vertebral body. Since L1 is readily identifiable on routine computed tomography, these ratios may provide a practical complementary anatomical reference. Further studies are warranted to validate their clinical applicability.

## 1. Introduction

The splenic artery (SA), originating from the celiac artery, is one of the most tortuous arteries in the body. SA extends behind the superior margin of the pancreas towards the spleen. Along its course, it gives off branches to the pancreas and stomach. Before entering the hilum of the spleen, SA gives off two or sometimes three branches, which divide into segmental branches (four or five) at the level of the hilum for supplying the spleen. Blood from the splenic tissue is collected by lobar veins, which unite to form the splenic vein (SV) within the splenorenal ligament. SV runs toward the midline to join the main portal vein, posterior to SA and the pancreatic tail and body [1].

The morphometrics of SA and SV have important clinical relevance, as their calibers may be affected by various congenital or acquired conditions, including liver cirrhosis and portal vein thrombosis [2,3,4]. Alterations in the diameter of SA and SV have traditionally been regarded as indicators of disturbances in portal or splanchnic hemodynamics [2,3,4]. For example, an SV diameter greater than 10 mm has been proposed as a practical anatomical criterion for predicting the occurrence of portal or SV thrombosis (PSVT) following laparoscopic splenectomy [2]. Moreover, an abnormal increase in the intraluminal diameter of SA, with a reported cut-off value exceeding 5.19 mm, has been associated with pathological conditions such as portal hypertension and cirrhosis [3]. Therefore, accurate recognition of SA and SV abnormalities, including dilatation or stenosis, requires that radiologists and surgeons be familiar with reference values for their diameters [2,3].

Demographic characteristics (sex, age, weight, height, body mass index, etc.) of subjects have been shown to influence the caliber of abdominal vessels such as SA [5,6]. For instance, previous studies have demonstrated a strong positive association between SA diameter and age in pediatric populations [6]. Given that anthropometric parameters—such as body surface area—may significantly affect vascular dimensions, some authors have proposed the use of alternative anatomical reference points, including the body of the first lumbar vertebra (L1), to establish a more standardized morphometric reference framework for the assessment of vascular pathologies such as stenosis or aneurysm formation [6,7,8]. Nevertheless, no studies to date have comprehensively evaluated the relationship between SA or SV caliber and L1 vertebral body across different age groups.

This retrospective examination was designed to uncover age-related ratios of SA and SV calibers to L1 and to provide CT-based morphometric reference data that may support the radiological assessment of portal and splanchnic hemodynamic disturbances.

## 2. Material and Methods

### 2.1. Ethical Statement

Ethical approval was obtained from the Institution’s Ethics Committee to conduct this retrospective computed tomography (CT) study (approval no: 2025/122012, date: 6 February 2025). All procedures followed were in accordance with the ethical standards of the responsible committee and with the Helsinki Declaration (as revised in 2013).

### 2.2. Study Design

The electronic hospital archive was retrospectively reviewed to identify patients aged 1–80 years who underwent routine contrast-enhanced abdominopelvic CT examinations. The electronic medical records included demographic information (sex and age), presenting complaints (e.g., abdominal pain), diagnostic and treatment records (laboratory findings, radiological examinations, clinical evaluations, medications, etc.), and hospital admission and discharge dates.

A study population with a homogeneous distribution in terms of age and sex was created to ensure a balanced study design and facilitate age- and sex-based comparisons. For this purpose, the following steps were followed in order.

Firstly, an a priori power analysis was performed using G*Power software version 3.1.9.7 (HHU, Düsseldorf, Germany) to determine the minimum sample size required for a one-way analysis of variance (ANOVA) comparing eight age-decade groups. Assuming an alpha level of 0.05, a statistical power of 80%, and a medium-to-large effect size (Cohen’s f = 0.30), the minimum required total sample size was estimated to be 168 participants (approximately 21 participants per group). Considering previous studies [6,7], each individual age group (1–80 years) was designed to include 10 subjects (five males and five females) to ensure a balanced distribution by age and sex. Accordingly, the final study sample consisted of 800 individuals (100 participants per age-decade group), substantially exceeding the minimum required sample size.

Secondly, considering the limited number of patients (especially pediatric cases) registered in the electronic hospital archive system, a two-month age limit was established to standardize the patients’ ages. For example, when determining the 1-year-old age group, subjects between 10–14 months were included in the group. A two-month age interval was selected to allow practical age stratification while maintaining sufficient eligible cases within each age group. This approach also enabled balanced sex distribution across individual age strata and reduced sampling imbalance caused by uneven archive availability.

Thirdly, a list of subjects aged 1–80 was created. The patients on the list were transferred to a document created for each age. Subjects in each document were distributed into two subgroups: male and female.

Fourthly, the electronic archive was reviewed retrospectively, starting from December 2024 and proceeding backwards. For each age (1–80 years), 10 individuals (5 males and 5 females) meeting the inclusion and exclusion criteria were consecutively selected. Once the predetermined number of subjects had been reached for a given age, no additional examinations from that age group were evaluated. This process was repeated for all ages and extended back to January 2020, resulting in a balanced study cohort of 800 subjects.

Lastly, the selected eligible cases were compiled into the final study cohort consisting of 800 subjects without detectable abdominopelvic pathology.

### 2.3. Inclusion and Exclusion Criteria

Subjects included in the study population were selected among patients who underwent contrast-enhanced abdominopelvic CT examinations for various clinical indications (e.g., minor trauma, pelvic pain, or abdominal pain) and were subsequently discharged without requiring medical or surgical treatment related to abdominopelvic, hepatobiliary, cardiovascular, or splenic vascular disorders. Eligibility was determined through evaluation of radiological findings, electronic medical records, and available clinical history. Only subjects with radiologically unremarkable abdominopelvic CT findings, no documented history of acute or chronic hepatobiliary, hematological, infectious, cardiovascular, or splenic vascular disease, and good-quality CT images were included in the analysis.

Exclusion criteria were as follows: (a) patients with a history of genetic disease, systemic disorder, congenital anomaly, portal hypertension, or any condition potentially affecting abdominopelvic, cardiovascular, or splenic vascular structures, (b) patients who had undergone surgical procedures involving abdominopelvic or cardiovascular structures (e.g., splenectomy, pancreatectomy, gastrectomy, or liver transplantation), (c) patients with any mass, lesion, cyst, or pathological condition involving the abdominopelvic region, spleen, or splenic vessels, and (d) patients with hemorrhage, free fluid, or free air within the abdominopelvic cavity.

### 2.4. Study Population

The 800 subjects (mean age: 40.50 ± 23.11 years), consisting of 400 males and 400 females, were included in this retrospective CT examination.

### 2.5. CT Protocol

A multidetector CT scanner was used to obtain the contrast-enhanced abdominopelvic CT images (Toshiba Alexion, TSX-034A, Tochigi, Japan). Patients were asked to fast for 4–6 h before the scan. In the majority of patients (over 6 years of age), the scans were carried out in the inspiratory phase. In some pediatric cases under 6 years of age, sedation was applied and thus the scans were carried out during free breathing. While the scans were being carried out, ALARA (as low as reasonably achievable) guidelines were taken into consideration; thus, appropriate milliampere-second values (mAs) and peak kilovoltage (kVp) were determined for each patient. The scan parameters were determined as follows: image matrix = 512 × 512, beam pitch = 1.00, field of view (FOV) = 30–40 cm, slice thickness = 1 mm, effective mAs = 80, and tube voltage = 120 kVp. A standard radiologic protocol was followed, and each patient received intravenous non-ionic iodinated contrast agent followed by physiological saline (between 20–30 mL). A 70 mL volume of contrast agent (Iohexol, 350 mg/mL; GE Healthcare, Cork, Ireland) was administered at a rate of 2–4 mL/s; the total volume did not exceed 80 mL. In children, the dose was calculated according to their body weight (1–2 mL per kilogram). Axial CT views were acquired, and then sagittal, coronal and three-dimensional reformatted views were reconstructed. The data were transferred to the radiological imaging system in the hospital (PACS, the picture archiving and communication system) and then analyzed via the Sectra Workstation IDS7 software (version 26.2; Sectra, Linköping, Sweden).

### 2.6. Morphometric Parameters

**Figure 1:**
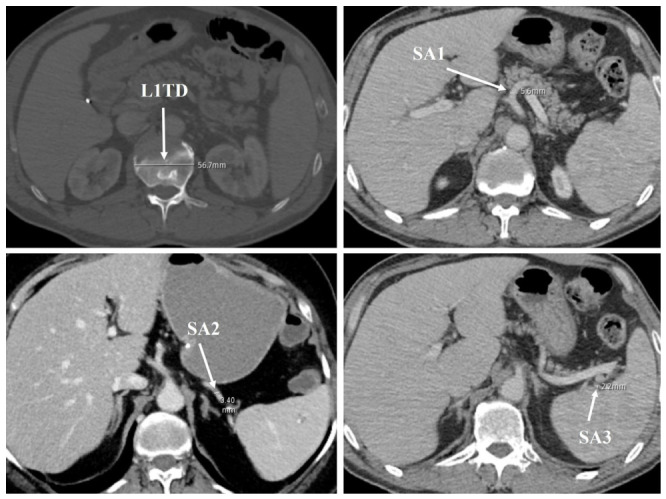
The parameters regarding L1 and the splenic artery. L1TD: the transverse diameter of L1’s body at its center, SA1: the arterial diameter measured at the proximal segment, 3 mm distal to its origin from the celiac trunk, SA2: the arterial diameter at the midpoint between the proximal and distal measurement sites, and SA3: the arterial diameter recorded at the distal segment, 3 mm proximal to the splenic hilum.

**Figure 2:**
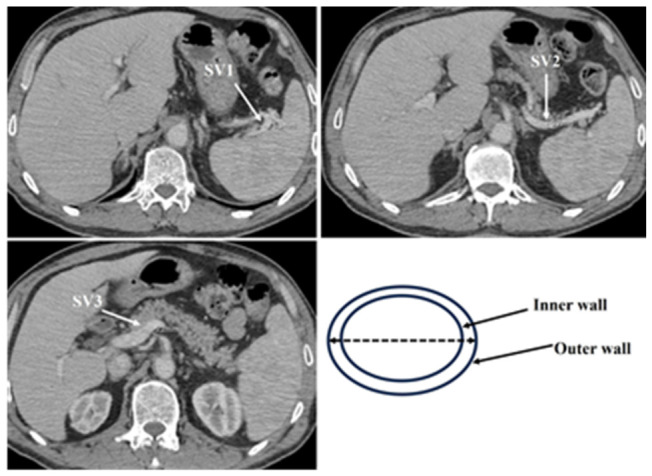
The parameters regarding the splenic vein. SV1: the venous diameter assessed at the proximal segment, 3 mm distal to the confluence of the splenic lobar veins; SV2: the venous diameter at the midpoint of the vein between its proximal and distal reference points; and SV3: the venous diameter measured at the distal segment, 3 mm before its junction with the main portal vein. The schematic diagram demonstrates the outer wall-to-outer wall measurement technique used for vascular diameter assessment. Measurements were performed perpendicular to the long axis of the vessel.

Six distinct measurements were obtained to evaluate the anatomical characteristics of SA and SV (Figure 1 and Figure 2). For SA, measurements included: (a) SA1, defined as the arterial diameter measured at the proximal segment, 3 mm distal to its origin from the celiac trunk; (b) SA2, the diameter at the midpoint between the proximal and distal measurement sites; and (c) SA3, the diameter recorded at the distal segment, 3 mm proximal to the splenic hilum. Correspondingly, SV measurements consisted of: (d) SV1, the venous diameter assessed at the proximal segment, 3 mm distal to the confluence of the splenic lobar veins; (e) SV2, the diameter at the midpoint of the vein between its proximal and distal reference points; and (f) SV3, the diameter measured at the distal segment, 3 mm before its junction with the main portal vein. All vascular diameters were obtained using oblique multiplanar reconstruction techniques. Measurements were performed on axial images oriented perpendicular to the longitudinal axis of each vessel to ensure accuracy. Due to technical challenges associated with identifying the intraluminal borders—particularly in infant cases—vascular diameters were measured from the outer wall to the outer wall of the vessel (Figure 2). This approach was adopted to maintain consistency and measurement standardization across all subjects.

The body of the first lumbar vertebra (L1) was selected as an internal anatomical reference. The transverse diameter of the L1 vertebral body (L1TD) was measured at its midpoint (Figure 1). The ratios of splenic vessel diameters to L1TD were subsequently calculated to serve as a practical anatomical reference.

### 2.7. Statistical Analysis

A radiologist (U.Y.) with seven years of experience measured all parameters. These measurements were taken into account to perform statistical evaluations. A second investigator (S.B.) measured the morphometric parameters twice in 160 individuals (two cases for each age, one male and one female, that is, 20% of the study population) to test repeatability of measurements. Interobserver agreement was assessed using the intraclass correlation coefficient (ICC) based on a two-way mixed-effects model with absolute agreement (single measures). ICC values with 95% confidence intervals (95% CIs) were calculated. The paired sample *t*-test was used to check the intra-observer reproducibility. Sex comparison was made using the independent samples *t*-test. Alterations in the parameters according to age (from one year to 80 years) were analyzed using One-way ANOVA. In addition, the subjects were divided into eight age decades (first: 1–10 years, second: 11–20 years, third: 21–30 years, fourth: 31–40 years, fifth: 41–50 years, sixth: 51–60 years, seventh: 61–70 years, and eighth: 71–80 years). Alterations in the parameters according to age decades (from first to eighth) were analyzed using One-way ANOVA. In light of Goodway et al.’s book [9], pediatric patients were divided into five age groups as follows: infancy: 1–2 years, early childhood: 3–5 years, late childhood: 6–9 years, prepubescent: 10–13 years, and postpubescent: 14–20 years. Changes in the parameters according to age groups (from the infancy period to the postpubescent period) were analyzed using One-way ANOVA. When the overall ANOVA demonstrated a statistically significant difference, Bonferroni-adjusted post hoc pairwise comparisons were performed to identify the specific groups responsible for the observed differences while controlling for multiple comparisons. Comparison of three SA-related measurements (SA1, SA2 and SA3) or three SV-related measurements (SV1, SV2 and SV3) was done using ANOVA with repeated measures. Correlations between SA1, SA2, SA3, SV1, SV2, SV3 and L1TD were assessed using the Pearson correlation coefficient test. Curvilinear or linear regression analysis was utilized to reveal growth dynamics of SA1, SA2, SA3, SV1, SV2, SV3 and L1TD in relation to age. The coefficient of determination (R^2^) was used to assess the match between the estimated functions and the numerical values. Levene’s test was used to evaluate homoscedasticity. The normality of the dataset was assessed using the Shapiro–Wilk test. This test was performed after dividing the study population into age groups. Parametric tests were used in statistical analyses, since the data were normally distributed. Using SPSS version 25.0 (IBM, Armonk, NY, USA), all analyses were performed by accepting “*p* < 0.05” as significant.

## 3. Results

### 3.1. Measurement Reliability

The intra-observer (*p* > 0.05) and inter-observer (ICC scores = 0.96–0.99 for the parameters, 95% CI: 0.947–0.997, *p* < 0.001) assessments demonstrated excellent measurement repeatability.

### 3.2. Age-Related Changes

Age-stratified measurements of SA and SV are summarized in Supplementary Table S1 (see Table S1 in the Supplementary Material). Additionally, age-based ratios of SA- and SV-related parameters to L1TD are presented in Supplementary Table S2 (see Table S2 in the Supplementary Material). The findings demonstrate a statistically significant association between age and the diameters of SA, SV, and the L1 vertebral body (*p* < 0.001).

When evaluated by age decades, L1TD demonstrated a progressive increase up to the early fifth decade, after which no statistically significant variation was observed. SV1 exhibited a proportional increase until the early fifth decade, followed by a decline beginning in the seventh decade. In contrast, SV2 and SV3 increased up to the early second decade and remained relatively stable thereafter, without statistically meaningful changes. SA1, SA2, and SA3 showed a gradual enlargement from 1 year of age through the early fifth decade, followed by a statistically significant reduction in later decades (Table 1).

Regarding normalized measurements, the ratios SV1/L1TD, SV2/L1TD, SV3/L1TD, SA1/L1TD, SA2/L1TD, and SA3/L1TD reached their maximum values during the first decade of life and demonstrated a statistically significant decrease in the second decade. Subsequently, these ratios generally followed a trend characterized by an initial increase and a subsequent decline with advancing age (Table 2).

When the analysis was restricted to pediatric age groups, spanning from infancy through the postpubescent period, L1TD demonstrated a proportional increase with age. SV1, SV2, and SV3 showed no significant variation from infancy through the prepubescent stage; however, all three parameters increased to a statistically significant extent during the postpubescent period. In contrast, SA1, SA2, and SA3 increased with a nonuniform pattern of change between 1 and 20 years of age (Table 3).

With respect to normalized values, SV1/L1TD, SV2/L1TD, SV3/L1TD, SA1/L1TD, SA2/L1TD, and SA3/L1TD generally demonstrated a downward trend as age advanced within the pediatric population (Table 4).

Adult measurements were greater than pediatric measurements (*p* < 0.001) (Table 5).

### 3.3. Sex-Related Comparisons

L1TD values were significantly higher in males compared with females (*p* < 0.001). In contrast, SV1, SV2, SV3, SA1, SA2, and SA3 did not demonstrate statistically significant differences between sexes (*p* > 0.05). Owing to the larger L1TD observed in males, the corresponding ratio values were significantly lower in males than in females (*p* < 0.001) (Table 6).

### 3.4. Correlation Analysis

Analysis revealed significant positive correlations across the evaluated morphometric parameters (Table 7). L1TD demonstrated a strong positive correlation with age. Among the vascular measurements, L1TD had a strongly positive correlation with SV1 and SV2, but a moderately positive correlation with SV3. L1TD had a weakly positive correlation with SA1, but a moderately positive correlation with SA2 and SA3.

### 3.5. Segmental Comparison of Splenic Vessel Diameters

SV diameters demonstrated a descending pattern from proximal to distal segments, with SV1 measuring the largest (5.21 ± 0.76 mm), followed by SV2 (4.35 ± 0.61 mm) and SV3 (3.43 ± 0.41 mm); these differences were statistically significant (*p* < 0.001). A similar trend was observed for SA, where SA1 showed the greatest diameter (2.75 ± 0.25 mm), followed by SA2 (2.43 ± 0.23 mm) and SA3 (2.09 ± 0.31 mm), also reaching statistical significance (*p* < 0.001). These findings suggest that neither SV nor SA exhibits a uniform cylindrical configuration; instead, both vessels demonstrate progressive tapering from their proximal to distal segments.

### 3.6. Regression Analysis

**Figure 3:**
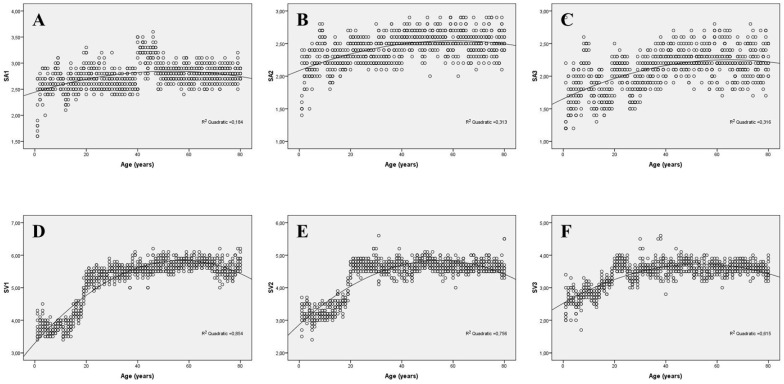
Quadratic functions. The fitted regression curves were included to provide a graphical illustration of age-related changes in the measured vascular parameters. Statistical analyses of age-group differences were performed separately using one-way ANOVA with Bonferroni-adjusted post hoc analyses. (**A**) SA1 (proximal splenic artery diameter), (**B**) SA2 (middle splenic artery diameter), (**C**) SA3 (distal splenic artery diameter), (**D**) SV1 (proximal splenic vein diameter), (**E**) SV2 (middle splenic vein diameter), and (**F**) SV3 (distal splenic vein diameter).

Quadratic regression analysis provided the best-fitting model for all evaluated parameters. The corresponding regression equations and coefficients of determination (R^2^) were presented below and illustrated in Figure 3. The quadratic regression equations for each parameter were as follows:

- L1TD = 29.256 + 0.747 × (Age) − 0.006440 × (Age)^2^ (R^2^: 0.663, *p* < 0.001)
- SV1 = 3.300 + 0.089 × (Age) − 0.000776 × (Age)^2^ (R^2^: 0.854, *p* < 0.001)
- SV2 = 2.867 + 0.072 × (Age) − 0.000658 × (Age)^2^ (R^2^: 0.756, *p* < 0.001)
- SV3 = 2.521 + 0.045 × (Age) − 0.000420 × (Age)^2^ (R^2^: 0.615, *p* < 0.001)
- SA1 = 2.444 + 0.015 × (Age) − 0.000143 × (Age)^2^ (R^2^: 0.184, *p* < 0.001)
- SA2 = 2.085 + 0.015 × (Age) − 0.000127 × (Age)^2^ (R^2^: 0.313, *p* < 0.001)
- SA3 = 1.653 + 0.018 × (Age) − 0.000142 × (Age)^2^ (R^2^: 0.316, *p* < 0.001)

## 4. Discussion

Our examination contributes to the literature regarding splenic vessel morphometry. To our knowledge, this is the first study to measure SA and SV diameters in a population standardized by sex and age, comprising individuals aged 1 to 80 years. It is also the first study to evaluate the relationship between SA/SV calibers and L1 across a broad age spectrum in order to provide supportive morphometric reference data for radiological assessment of splenic vascular abnormalities. Moreover, our regression equations may be useful for estimating age-specific SA and SV diameters.

Alterations in vessel calibers are of great importance for surgeons and radiologists for detecting pathologies like aneurysm or stenosis [2,3]. For example, an artery or vein that is dilated more than 1.5 times its expected caliber is a sign of an aneurysm [10,11,12]. Although this cut-off value allows the diagnosis of pathological entities like vascular dilation, clinicians should remember that vascular calibers are affected by various factors such as body shape, body mass index, weight, height, sex, age, diet, or race [2,3,6]. Therefore, some investigators have used anthropometric indicators (e.g., body surface area) to establish a comprehensive standard for detecting alterations in vessel diameters [13]. For example, the caliber of the main portal vein correlated positively with demographic features of patients (age, height, weight, body surface area, etc.) [13]. However, some clinicians have raised concerns regarding the accuracy of anthropometric measurements obtained in emergency settings, particularly in infants and toddlers [14]. To evaluate the relationship between body size and vessel diameters, some investigators recommend using L1TD as the dorsal cut-off to trace L1’s body [6,7,8]. This recommendation is supported by the following considerations: (a) any variation in L1’s orientation has a minor effect on caliber and area outcomes, (b) precursor cross-sectional imaging investigations focusing on organ volumes’ calculation display that normalization of data according to L1-based indices accounted for body habitus, (c) L1 is quite easy to find on abdominal radiological sections, and (d) L1 is visible on almost all abdominopelvic CT images [15]. Thus, L1TD may serve as a practical anatomical reference that can complement the assessment of SA and SV calibers on routine CT examinations.

Some pathologies associated with splenic vessels may reach high morbidity and mortality rates [16]. For instance, SA aneurysms account for approximately 60% of all splanchnic artery aneurysms; thus, they are considered the most common type of visceral aneurysm [17]. Spontaneous ruptures, seen in 2–10% of patients, are the most dangerous complication and have a mortality rate of up to 25% [18,19]. This mortality rate increases up to 75% during pregnancy [20]. SV aneurysm, a rare condition, is often associated with portal hypertension [21]. Shimoda et al. [21] successfully treated a 72-year-old male with abdominal hemorrhage, distention, and pain due to SV aneurysm rupture. Cekirge et al. [22] encountered SV stenosis in a 56-year-old male during transjugular intrahepatic portosystemic shunt placement on account of gastric variceal bleeding. A fundamental requirement for radiologists and surgeons to recognize splenic vessel abnormalities, such as narrowing or dilatation, is a thorough understanding of reference values for SA and SV calibers [2,3]. Thus, the morphometrics of SA and SV have clinical implications. For example, the optimal cut-off value of abnormal intraluminal SA caliber to predict portal hypertension or cirrhosis was reported by Zeng et al. [3] as >5.19 mm. A cut-off value of >4 mm for predicting SA steal syndrome after liver transplantation was reported by Kirbas et al. [23]. Furthermore, cut-off values of >10 mm and >8 mm for predicting PSVT after laparoscopic splenectomy were reported by Kuroki et al. [2] and Danno et al. [24], respectively. For predicting portal vein thrombosis after splenectomy, cut-off values of >14 mm and >9 mm were reported by Kuroki et al. [2] and Kinjo et al. [25], respectively. Familiarity with reference values for SA and SV calibers is clinically relevant for several purposes: (a) identifying splenic vessel anomalies, (b) preoperative evaluation and postoperative follow-up after liver transplantation or splenectomy, and (c) assessing portal hypertension [2,3,26]. In this context, the quadratic functions proposed in our study may serve as a useful tool for estimating age-specific calibers of SA and SV across the lifespan. Nevertheless, although the present study provides comprehensive age-specific morphometric reference values and regression equations for SA and SV calibers, their clinical applicability and diagnostic performance should be validated in future studies involving patients with splenic vascular disorders.

In the pediatric group, the diameters of SV1, SV2, SV3, SA1, SA2, and SA3 were 4 ± 0.45 mm, 3.39 ± 0.39 mm, 2.88 ± 0.37 mm, 2.60 ± 0.26 mm, 2.23 ± 0.26 mm, and 1.82 ± 0.33 mm, respectively. Among adults, these values were 5.61 ± 0.23 mm, 4.68 ± 0.19 mm, 3.61 ± 0.22 mm, 2.80 ± 0.22 mm, 2.49 ± 0.18 mm, and 2.18 ± 0.24 mm, respectively. All pediatric measurements were significantly smaller than those in adults (*p* < 0.001). In our examination, SV1 exhibited a proportional increase until the early fifth decade, followed by a decline beginning in the seventh decade. In contrast, SV2 and SV3 increased up to the early second decade and remained relatively stable thereafter, without statistically meaningful changes. SA1, SA2, and SA3 showed a gradual enlargement from 1 year of age through the early fifth decade, followed by a statistically significant reduction in later decades. These findings indicate that splenic vessel calibers should be interpreted in an age-specific context, as a single reference threshold may not be appropriate across the entire lifespan. In this regard, the age-specific observed ranges of SA and SV diameters may provide context for the interpretation of splenic vessel-related abnormalities, such as PSVT. Consistent with previous studies [6], we found that the caliber of SA did not differ significantly between males and females. Thus, our age-specific observed ranges may provide interpretative context for both sexes. Moreover, we noted that SA and SV diameters decrease from the proximal to the distal segment, indicating that SA or SV does not have a cylindrical shape. Measurements obtained from different segments of the same vessel should not be considered interchangeable during radiological assessment. For this reason, we suggest that the distinct values observed for the proximal, middle, and distal segments of SA and SV should be considered when evaluating splenic vessel-related anomalies. On the other hand, correlation analysis demonstrated that L1TD was strongly correlated with age and showed consistently stronger correlations with splenic venous calibers than with splenic arterial calibers. Since chronological age also demonstrated stronger correlations with splenic venous than with splenic arterial calibers, the stronger associations observed between L1TD and venous measurements may, at least in part, be explained by their shared relationship with age. Values that fall outside the observed ranges presented in supplementary digital Tables S1 and S2 (see Tables S1 and S2 in the Supplementary Materials) may raise suspicion of pathological conditions, including stenosis or aneurysm of SA and SV; however, they should be evaluated together with clinical and radiological findings, and their diagnostic performance should be established in future studies involving pathological cohorts. Aktürk and Gunes [8] reported the ratio of the proximal abdominal aorta to L1 as 0.41 in pediatric subjects younger than 133 months (a period during which L1TD was similar in both sexes), and therefore suggested that a ratio exceeding 0.4 might indicate abdominal aortic dilation. Based on this information, we consider that the relationship between SA/SV and L1 may also provide context for the interpretation of splenic vessel-related abnormalities. For instance, we calculated SA2/L1TD ratio to be 0.05 (observed range: 0.04–0.11) and SV2/L1TD ratio to be 0.10 (observed range: 0.07–0.17) in individuals aged 1 to 80 years, and thus considered that values falling outside these observed ranges may raise suspicion of abnormal calibers associated with narrowing or dilatation in the mid-portions of SA and SV and should be evaluated together with clinical and radiological findings. Since L1 can be easily visualized on routine abdominopelvic CT images, SA-to-L1 and SV-to-L1 ratios may provide supplementary morphometric information during radiological evaluation of splenic vascular abnormalities [27]. Further studies evaluating these ratios in pathological cohorts are needed to better define their potential clinical applicability.

The concept of vertebra-based normalization has emerged as a valuable method for assessing vascular structures. For instance, Akay et al. [28] demonstrated that ratios between thoracic vascular diameters and the anteroposterior diameter of a thoracic vertebral body remained relatively constant across pediatric age groups, despite significant age-related increases in vessel calibers. Based on these findings, the authors suggested that vertebral measurements may serve as practical internal anatomical references during radiological assessment of pediatric vascular structures [28]. This concept has also been applied to abdominal arterial and venous structures, particularly using the L1 as an internal anatomical reference. For example, Aktürk and Gunes [8] utilized the L1TD to standardize abdominal aortic measurements in pediatric patients, particularly in situations where anthropometric indicators such as body surface area were unavailable or difficult to obtain. Likewise, Beger and Ten [6] evaluated age-dependent changes in abdominal visceral artery calibers and their ratios to L1 in children, suggesting that L1-based normalization may contribute to the establishment of supportive morphometric standards during radiological assessment of vascular abnormalities. More recently, Binici et al. [27] investigated the relationship between portal vein calibers and L1 in subjects aged 1–80 years and reported that age-specific vessel-to-L1 ratios may provide useful reference data for evaluating portal venous disorders. Unlike external anthropometric parameters such as height, weight, body mass index, or body surface area, L1TD can be measured directly from the same CT examination without requiring additional clinical information [6,27]. Because vertebral body size generally reflects overall body habitus and skeletal growth [15], L1TD may provide a practical internal anatomical reference for vascular normalization [6,7,8,27]. This approach may be particularly advantageous in emergency settings, where accurate anthropometric measurements are often unavailable, incomplete, or difficult to obtain. Therefore, L1-based normalization should be regarded as a practical complementary method for CT-based vascular assessment rather than a replacement for conventional anthropometric parameters. Building on these previous studies, the present study extends the concept of L1-based normalization to SA and SV calibers across a broad age spectrum and provides age-specific CT-based reference values that may serve as supportive reference data during the assessment of splenic vascular abnormalities.

This work possesses some limitations. Firstly, our sample size is moderate. In further investigations, using a study population with a larger sample size may be beneficial for a more precise understanding of age-related change in SA or SV diameter. Secondly, only L1TD was used as the anthropometric indicator in our study. The study was designed as a retrospective, and thus it was not possible to access demographic data of some individuals (e.g., their heights, weights, or body mass indices). Although L1TD may provide a practical internal anatomical reference, it should not be considered a complete substitute for conventional anthropometric parameters such as height, weight, body mass index, or body surface area. Thus, further retrospective or prospective investigations including alternative indicators (body surface area, etc.) may be beneficial for radiologists and surgeons to understand the growth pattern of SA or SV across the lifespan. Thirdly, two-dimensional CT views were used to measure the parameters. Further examinations, including alternative methodologies like measurements on three-dimensional CT views, may allow for a better understanding of the parameters regarding SA or SV diameter. Fourthly, morphometric analyses were performed using radiological reconstructions rather than direct anatomical specimens. Therefore, image resolution limitations and minor software-related processing inaccuracies may have partially influenced the linear measurements, particularly in young children. Fifthly, all vascular diameters in the present study were measured using the outer wall-to-outer wall technique, as accurate delineation of the intraluminal borders was not always feasible, particularly in younger children. Therefore, the age-specific reference values presented herein should not be directly compared with published luminal (inner wall-to-inner wall) cut-off values unless the same measurement methodology has been used. Sixthly, although all examinations were performed according to the institution’s routine standardized CT protocol, the retrospective inclusion of examinations acquired between 2020 and 2024 may have introduced minor variability related to scanner software updates or reconstruction algorithms, which could not be completely excluded. Lastly, the study population was derived from patients who underwent abdominopelvic CT examinations for various clinical indications, including pelvic pain, minor trauma, and abdominal pain. According to electronic medical records and hospital follow-up data, these individuals were discharged without requiring medical treatment affecting abdominopelvic or cardiovascular structures, or surgical interventions such as splenectomy, pancreatectomy, gastrectomy, or liver transplantation. Subjects were considered eligible for inclusion when no clinically significant abdominopelvic, hepatobiliary, cardiovascular, or splenic vascular pathology requiring medical or surgical management was identified during clinical evaluation, radiological assessment, or follow-up. Nevertheless, due to the retrospective, single-center, and hospital-based design of the study, the possibility of residual selection bias or undetected subclinical conditions cannot be completely excluded. Therefore, our findings should be interpreted as CT-based reference data obtained from individuals without detectable abdominopelvic pathology and should not be regarded as values derived from a healthy population. In addition, obtaining CT images from asymptomatic healthy volunteers—particularly in pediatric populations—is ethically and practically challenging because ionizing radiation exposure cannot be justified without clinical indication. Despite these limitations, we believe that the present study provides valuable age-specific morphometric reference data regarding SA and SV calibers and their relationship with L1 across a broad age spectrum.

## 5. Conclusions

The age-related caliber measurements and derived SA- and SV-to-L1 ratios presented in this study may provide supportive morphometric reference data for the radiological assessment of splenic vascular structures across different age groups. Because the L1 vertebral body is consistently identifiable on routine abdominopelvic CT examinations, the proposed ratios may serve as a practical complementary anatomical reference for morphometric evaluation. Nevertheless, their potential clinical applicability should be confirmed in future studies including patients with splenic vascular disorders.

## Figures and Tables

**Table 1:** Changes in the parameters according to decades.

Decades	N	L1TD (mm)	SV1 (mm)	SV2 (mm)	SV3 (mm)	SA1 (mm)	SA2 (mm)	SA3 (mm)
1st	100	30.22 ± 5.47 ^a,b,c,d,e,f,g^	3.78 ± 0.21 ^a,b,c,d,e,f,g^	3.19 ± 0.24 ^a,b,c,d,e,f,g^	2.71 ± 0.32 ^a,b,c,d,e,f,g^	2.58 ± 0.29 ^b,d,e,f,g^	2.22 ± 0.31 ^b,c,d,e,f,g^	1.86 ± 0.39 ^c,d,e,f,g^
2nd	100	43.09 ± 3.76 ^b,c,d,e,f,g^	4.22 ± 0.51 ^b,c,d,e,f,g^	3.58 ± 0.41 ^b,c,d,e,f,g^	3.06 ± 0.33 ^b,c,d,e,f,g^	2.62 ± 0.23 ^d,e,f,g^	2.25 ± 0.19 ^b,c,d,e,f,g^	1.78 ± 0.25 ^b,c,d,e,f,g^
3rd	100	45.97 ± 4.00 ^d,e,f,g^	5.39 ± 0.19 ^c,d,e,f,g^	4.72 ± 0.16	3.57 ± 0.28	2.71 ± 0.18 ^d^	2.38 ± 0.15 ^d,e,f,g^	1.96 ± 0.25 ^c,d,e,f,g^
4th	100	46.92 ± 3.73 ^e,f,g^	5.53 ± 0.20 ^d,e,f,g^	4.64 ± 0.22	3.63 ± 0.26	2.66 ± 0.21 ^d,e,f,g^	2.42 ± 0.18 ^d,e,f,g^	2.13 ± 0.20 ^d,e^
5th	100	48.27 ± 3.41 ^g^	5.65 ± 0.21 ^g^	4.68 ± 0.21	3.68 ± 0.19	3.07 ± 0.25 ^e,f,g^	2.57 ± 0.14 ^f,g^	2.28 ± 0.17 ^f,g^
6th	100	49.44 ± 3.99	5.73 ± 0.16 ^g^	4.68 ± 0.17	3.58 ± 0.20	2.79 ± 0.14	2.55 ± 0.16	2.26 ± 0.21
7th	100	49.52 ± 3.86	5.77 ± 0.15 ^g^	4.66 ± 0.17	3.61 ± 0.17	2.78 ± 0.16	2.53 ± 0.19	2.23 ± 0.24
8th	100	50.66 ± 4.22	5.60 ± 0.25	4.68 ± 0.20	3.61 ± 0.17	2.79 ± 0.14	2.52 ± 0.18	2.23 ± 0.24
*p*	800	<0.001	<0.001	<0.001	<0.001	<0.001	<0.001	<0.001

Data are presented as a mean ± standard deviation ^a^: Comparison to 2nd decade, ^b^: Comparison to 3rd decade, ^c^: Comparison to 4th decade, ^d^: Comparison to 5th decade, ^e^: Comparison to 6th decade, ^f^: Comparison to 7th decade, ^g^: Comparison to 8th decade, SV1: diameter of the splenic vein at the proximal level, SV2: diameter of the splenic vein at the middle level, SV3: diameter of the splenic vein at the distal level, SA1: diameter of the splenic artery at the proximal level, SA2: diameter of the splenic artery at the middle level, SA3: diameter of the splenic artery at the distal level, L1TD: transverse diameter of L1’s body, *p* < 0.05 statistically significant.

**Table 2:** Changes in the ratios according to decades.

Decades	N	SV1/L1TD	SV2/L1TD	SV3/L1TD	SA1/L1TD	SA2/L1TD	SA3/L1TD
1st	100	0.13 ± 0.03 ^a,b,c,d,e,f,g^	0.11 ± 0.02 ^a,b,c,d,e,f,g^	0.09 ± 0.02 ^a,b,c,d,e,f,g^	0.09 ± 0.01 ^a,b,c,d,e,f,g^	0.07 ± 0.01 ^a,b,c,d,e,f,g^	0.06 ± 0.01 ^a,b,c,d,e,f,g^
2nd	100	0.10 ± 0.01 ^b,c,d,e,f,g^	0.08 ± 0.01 ^b,c,d,e,f,g^	0.07 ± 0.01 ^b,c,d^	0.06 ± 0.01 ^c,e,f,g^	0.05 ± 0.01	0.04 ± 0.01 ^c,d,e,f^
3rd	100	0.12 ± 0.01 ^g^	0.10 ± 0.01 ^d,e,f,g^	0.08 ± 0.01 ^e,f,g^	0.06 ± 0.01 ^d,g^	0.05 ± 0.01	0.04 ± 0.01 ^d^
4th	100	0.12 ± 0.01 ^g^	0.10 ± 0.01 ^g^	0.08 ± 0.01 ^e,f,g^	0.06 ± 0.01 ^d^	0.05 ± 0.01	0.05 ± 0.01
5th	100	0.12 ± 0.01 ^g^	0.10 ± 0.01	0.08 ± 0.01 ^g^	0.06 ± 0.01 ^e,f,g^	0.05 ± 0.01 ^g^	0.05 ± 0.00 ^g^
6th	100	0.12 ± 0.01	0.10 ± 0.01	0.07 ± 0.01	0.06 ± 0.01	0.05 ± 0.01	0.05 ± 0.01
7th	100	0.12 ± 0.01	0.09 ± 0.01	0.07 ± 0.01	0.06 ± 0.01	0.05 ± 0.01	0.05 ± 0.01
8th	100	0.11 ± 0.01	0.09 ± 0.01	0.07 ± 0.01	0.06 ± 0.01	0.05 ± 0.01	0.04 ± 0.01
*p*	800	<0.001	<0.001	<0.001	<0.001	<0.001	<0.001

Data are presented as a mean ± standard deviation ^a^: Comparison to 2nd decade, ^b^: Comparison to 3rd decade, ^c^: Comparison to 4th decade, ^d^: Comparison to 5th decade, ^e^: Comparison to 6th decade, ^f^: Comparison to 7th decade, ^g^: Comparison to 8th decade, SV1: diameter of the splenic vein at the proximal level, SV2: diameter of the splenic vein at the middle level, SV3: diameter of the splenic vein at the distal level, SA1: diameter of the splenic artery at the proximal level, SA2: diameter of the splenic artery at the middle level, SA3: diameter of the splenic artery at the distal level, L1TD: transverse diameter of L1’s body, *p* < 0.05 statistically significant.

**Table 3:** Changes in the parameters according to pediatric age groups.

Pediatric Age Groups	N	L1TD (mm)	SV1 (mm)	SV2 (mm)	SV3 (mm)	SA1 (mm)	SA2 (mm)	SA3 (mm)
Infancy	20	23.24 ± 1.78 ^a,b,c,d^	3.77 ± 0.26 ^d^	3.20 ± 0.29 ^d^	2.58 ± 0.37 ^d^	2.28 ± 0.40 ^a,b,c,d^	1.91 ± 0.34 ^a,b,c,d^	1.60 ± 0.44 ^b^
Early childhood	30	27.33 ± 1.93 ^b,c,d^	3.82 ± 0.25 ^d^	3.19 ± 0.30 ^d^	2.65 ± 0.32 ^d^	2.57 ± 0.21	2.13 ± 0.21 ^b,d^	1.69 ± 0.25 ^b^
Late childhood	40	33.92 ± 3.04 ^c,d^	3.72 ± 0.16 ^d^	3.18 ± 0.18 ^d^	2.76 ± 0.29 ^d^	2.71 ± 0.20 ^c^	2.39 ± 0.24 ^c^	2.08 ± 0.33 ^c,d^
Prepubescent	40	39.86 ± 3.25 ^d^	3.79 ± 0.20 ^d^	3.27 ± 0.19 ^d^	2.78 ± 0.19 ^d^	2.50 ± 0.20 ^d^	2.20 ± 0.22	1.81 ± 0.28
Postpubescent	70	44.21 ± 3.40	4.41 ± 0.47	3.71 ± 0.42	3.20 ± 0.27	2.70 ± 0.21	2.30 ± 0.17	1.80 ± 0.26
*p*		<0.001	<0.001	<0.001	<0.001	<0.001	<0.001	<0.001

Data are presented as a mean ± standard deviation, ^a^: Comparison to early childhood, ^b^: Comparison to late childhood, ^c^: Comparison to prepubescent, ^d^: Comparison to postpubescent, SV1: diameter of the splenic vein at the proximal level, SV2: diameter of the splenic vein at the middle level, SV3: diameter of the splenic vein at the distal level, SA1: diameter of the splenic artery at the proximal level, SA2: diameter of the splenic artery at the middle level, SA3: diameter of the splenic artery at the distal level, L1TD: transverse diameter of L1’s body, *p* < 0.05 statistically significant.

**Table 4:** Changes in the ratios according to pediatric age groups.

Pediatric Age Groups	N	SV1/L1TD	SV2/L1TD	SV3/L1TD	SA1/L1TD	SA2/L1TD	SA3/L1TD
Infancy	20	0.16 ± 0.02 ^a,b,c,d^	0.14 ± 0.02 ^a,b,c,d^	0.11 ± 0.02 ^a,b,c,d^	0.10 ± 0.02 ^b,c,d^	0.08 ± 0.01 ^b,c,d^	0.07 ± 0.02 ^b,c,d^
Early childhood	30	0.14 ± 0.01 ^b,c,d^	0.12 ± 0.01 ^b,c,d^	0.10 ± 0.01 ^b,c,d^	0.09 ± 0.01 ^b,c,d^	0.08 ± 0.01 ^b,c,d^	0.06 ± 0.01 ^c,d^
Late childhood	40	0.11 ± 0.01 ^c,d^	0.09 ± 0.01 ^c,d^	0.08 ± 0.01 ^c,d^	0.08 ± 0.01 ^c,d^	0.07 ± 0.01 ^c,d^	0.06 ± 0.01 ^c,d^
Prepubescent	40	0.10 ± 0.01	0.08 ± 0.01	0.07 ± 0.01	0.06 ± 0.01	0.06 ± 0.01	0.05 ± 0.01
Postpubescent	70	0.10 ± 0.01	0.08 ± 0.01	0.07 ± 0.01	0.06 ± 0.01	0.05 ± 0.01	0.04 ± 0.01
*p*	200	<0.001	<0.001	<0.001	<0.001	<0.001	<0.001

Data are presented as a mean ± standard deviation, For children: ^a^: Comparison to early childhood, ^b^: Comparison to late childhood, ^c^: Comparison to prepubescent, ^d^: Comparison to postpubescent, SV1: diameter of the splenic vein at the proximal level, SV2: diameter of the splenic vein at the middle level, SV3: diameter of the splenic vein at the distal level, SA1: diameter of the splenic artery at the proximal level, SA2: diameter of the splenic artery at the middle level, SA3: diameter of the splenic artery at the distal level, L1TD: transverse diameter of L1’s body, *p* < 0.05 statistically significant.

**Table 5:** Comparison of the parameters in children and adults.

Parameters	Children (*n* = 200)	Adults (*n* = 600)	*p*
L1TD (mm)	36.65 ± 7.97	48.46 ± 4.18	<0.001
SV1 (mm)	4.00 ± 0.45	5.61 ± 0.23	<0.001
SV2 (mm)	3.39 ± 0.39	4.68 ± 0.19	<0.001
SV3 (mm)	2.88 ± 0.37	3.61 ± 0.22	<0.001
SA1 (mm)	2.60 ± 0.26	2.80 ± 0.22	<0.001
SA2 (mm)	2.23 ± 0.26	2.49 ± 0.18	<0.001
SA3 (mm)	1.82 ± 0.33	2.18 ± 0.24	<0.001

Data are presented as a mean ± standard deviation, SV1: diameter of the splenic vein at the proximal level, SV2: diameter of the splenic vein at the middle level, SV3: diameter of the splenic vein at the distal level, SA1: diameter of the splenic artery at the proximal level, SA2: diameter of the splenic artery at the middle level, SA3: diameter of the splenic artery at the distal level, L1TD: transverse diameter of L1’s body, *p* < 0.05 statistically significant.

**Table 6:** Comparison of the parameters in terms of sex.

Parameters	Females (*n* = 400)	Males (*n* = 400)	*p*
L1TD (mm)	43.73 ± 6.73	47.29 ± 7.67	<0.001
SV1 (mm)	5.20 ± 0.77	5.21 ± 0.75	0.900
SV2 (mm)	4.37 ± 0.62	4.34 ± 0.61	0.508
SV3 (mm)	3.44 ± 0.42	3.42 ± 0.40	0.701
SA1 (mm)	2.75 ± 0.26	2.74 ± 0.24	0.480
SA2 (mm)	2.43 ± 0.24	2.43 ± 0.23	0.903
SA3 (mm)	2.10 ± 0.31	2.08 ± 0.31	0.486
SV1/L1TD	0.12 ± 0.01	0.11 ± 0.01	<0.001
SV2/L1TD	0.10 ± 0.01	0.09 ± 0.01	<0.001
SV3/L1TD	0.08 ± 0.01	0.07 ± 0.01	<0.001
SA1/L1TD	0.06 ± 0.01	0.06 ± 0.01	<0.001
SA2/L1TD	0.06 ± 0.01	0.05 ± 0.01	<0.001
SA3/L1TD	0.05 ± 0.01	0.05 ± 0.01	<0.001

Data are presented as a mean ± standard deviation, SV1: diameter of the splenic vein at the proximal level, SV2: diameter of the splenic vein at the middle level, SV3: diameter of the splenic vein at the distal level, SA1: diameter of the splenic artery at the proximal level, SA2: diameter of the splenic artery at the middle level, SA3: diameter of the splenic artery at the distal level, L1TD: transverse diameter of L1’s body, *p* < 0.05 statistically significant.

**Table 7:** Correlations between the parameters.

Parameters	L1TD	SV1	SV2	SV3	SA1	SA2	SA3
**Age**	0.701 **	0.786 **	0.703 **	0.615 **	0.331 **	0.496 **	0.518 **
	<0.001	<0.001	<0.001	<0.001	<0.001	<0.001	<0.001
**L1TD**		0.732 **	0.702 **	0.668 **	0.342 **	0.474 **	0.431 **
		<0.001	<0.001	<0.001	<0.001	<0.001	<0.001
**SV1**			0.944 **	0.826 **	0.409 **	0.538 **	0.541 **
			<0.001	<0.001	<0.001	<0.001	<0.001
**SV2**				0.853 **	0.397 **	0.520 **	0.520 **
				<0.001	<0.001	<0.001	<0.001
**SV3**					0.477 **	0.565 **	0.557 **
					<0.001	<0.001	<0.001
**SA1**						0.795 **	0.682 **
						<0.001	<0.001
**SA2**							0.890 **
							<0.001

SV1: diameter of the splenic vein at the proximal level, SV2: diameter of the splenic vein at the middle level, SV3: diameter of the splenic vein at the distal level, SA1: diameter of the splenic artery at the proximal level, SA2: diameter of the splenic artery at the middle level, SA3: diameter of the splenic artery at the distal level, L1TD: transverse diameter of L1’s body, **: *p* < 0.01 statistically significant.

## Data Availability

Available with the corresponding author on reasonable request.

## Supplementary Materials

The following supporting information can be downloaded at: https://www.mdpi.com/article/10.3390/diagnostics16142267/s1, Table S1: Age-dependent changes in diameters of the splenic vein and splenic artery from the age of 1 year to 80 years; Table S2: Ratios of the parameters to L1TD in all ages.

## Abbreviations

The following abbreviations are used in this manuscript:

SA	Splenic artery
SV	Splenic vein
PSVT	Portal or splenic vein thrombosis
L1	First lumbar vertebra
CT	Computed tomography
ALARA	As low as reasonably achievable
mAs	Milliampere-second values
kVp	Peak kilovoltage
PACS	The picture archiving and communication system
ICC	Intra-class correlation coefficients
L1TD	The transverse diameter of the first lumbar vertebra’s body at its center
SA1	Proximal splenic artery diameter
SA2	Middle splenic artery diameter
SA3	Distal splenic artery diameter
SV1	Proximal splenic vein diameter
SV2	Middle splenic vein diameter
SV3	Distal splenic vein diameter
