# Supplementary material for: CT-Based Reference Values for Splenic Artery and Vein Diameters in Individuals Aged 1–80 Years
Source: Diagnostics (Basel). 2026 Jul 20;16(14):2267. doi: 10.3390/diagnostics16142267 (PMC13408975; doi:10.3390/diagnostics16142267)
Supplement: Supplementary file 1 [file diagnostics-16-02267-s001.zip › diagnostics-4350378-supplementary.pdf]

Supplemental Digital Tables

Supplemental Digital Table 1: Age-dependent changes in diameters of the splenic vein and splenic artery from the age of 1 year to 80 years

Age	SV1 (mm)	SV2 (mm)	SV3 (mm)	SA1 (mm)	SA2 (mm)	SA3 (mm)
1	3.75±0.32 (3.40-4.30)	3.19±0.38 (2.50-3.70)	2.43±0.45 (2.00-3.40)	2.05±0.44 (1.60-2.70)	1.76±0.38 (1.40-2.40)	1.56±0.59 (1.20-2.90)
2	3.78±0.21 (3.50-4.10)	3.21±0.20 (3.00-3.50)	2.72±0.17 (2.50-3.00)	2.51±0.16 (2.30-2.80)	2.06±0.21 (1.80-2.40)	1.64±0.23 (1.40-2.00)
3	3.98±0.28 (3.60-4.50)	3.37±0.27 (2.90-3.70)	2.74±0.36 (2.00-3.30)	2.60±0.21 (2.20-2.90)	2.14±0.16 (1.80-2.30)	1.57±0.15 (1.40-1.80)
4	3.78±0.18 (3.50-4.00)	3.28±0.16 (3.00-3.50)	2.76±0.22 (2.50-3.10)	2.57±0.26 (2.00-2.90)	2.13±0.28 (1.50-2.40)	1.81±0.31 (1.20-2.20)
5	3.70±0.19 (3.50-4.00)	2.93±0.26 (2.40-3.30)	2.44±0.27 (2.00-2.90)	2.55±0.17 (2.40-2.90)	2.11±0.21 (1.80-2.50)	1.68±0.21 (1.40-2.00)
6	3.67±0.19 (3.50-4.10)	3.09±0.22 (2.80-3.50)	2.60±0.14 (2.40-2.80)	2.57±0.11 (2.40-2.70)	2.22±0.14 (2.00-2.40)	1.85±0.18 (1.60-2.10)
7	3.63±0.08 (3.50-3.80)	3.14±0.13 (3.00-3.40)	2.54±0.34 (1.70-2.90)	2.57±0.12 (2.40-2.80)	2.26±0.16 (2.00-2.50)	1.82±0.27 (1.40-2.20)
8	3.75±0.13 (3.60-3.90)	3.23±0.16 (3.00-3.50)	2.91±0.20 (2.70-3.30)	2.79±0.17 (2.50-3.00)	2.48±0.25 (2.00-2.80)	2.24±0.24 (1.80-2.60)
9	3.84±0.13 (3.70-4.00)	3.25±0.18 (3.00-3.60)	2.99±0.19 (2.70-3.20)	2.89±0.18 (2.60-3.10)	2.61±0.14 (2.40-2.80)	2.41±0.12 (2.20-2.50)
10	3.90±0.08 (3.80-4.00)	3.22±0.18 (3.00-3.50)	2.92±0.15 (2.70-3.20)	2.66±0.13 (2.50-2.80)	2.43±0.16 (2.20-2.60)	2.06±0.28 (1.60-2.50)
11	3.63±0.19 (3.40-4.00)	3.20±0.15 (3.00-3.50)	2.81±0.15 (2.60-3.10)	2.58±0.11 (2.40-2.70)	2.24±0.13 (2.00-2.40)	1.85±0.19 (1.50-2.10)
12	3.84±0.22 (3.50-4.10)	3.36±0.20 (3.10-3.60)	2.68±0.15 (2.50-2.90)	2.28±0.12 (2.10-2.50)	1.98±0.11 (1.80-2.20)	1.64±0.15 (1.40-1.90)
13	3.79±0.19 (3.50-4.00)	3.28±0.20 (3.00-3.60)	2.69±0.19 (2.40-3.00)	2.48±0.18 (2.20-2.80)	2.14±0.19 (1.90-2.40)	1.70±0.28 (1.30-2.00)
14	3.82±0.18 (3.50-4.10)	3.35±0.18 (3.10-3.60)	2.88±0.13 (2.70-3.10)	2.74±0.15 (2.50-3.00)	2.37±0.16 (2.10-2.60)	1.92±0.17 (1.60-2.10)
15	4.17±0.23 (3.80-4.50)	3.60±0.22 (3.20-3.90)	3.18±0.18 (3.00-3.50)	2.58±0.10 (2.40-2.70)	2.29±0.10 (2.20-2.50)	1.77±0.22 (1.50-2.10)
16	4.34±0.16 (4.10-4.60)	3.44±0.10 (3.30-3.60)	3.04±0.11 (2.90-3.20)	2.59±0.18 (2.30-2.90)	2.23±0.17 (2.00-2.50)	1.58±0.14 (1.40-1.80)
17	4.19±0.22 (3.90-4.60)	3.51±0.21 (3.10-3.80)	3.15±0.20 (2.80-3.40)	2.62±0.15 (2.40-2.80)	2.20±0.09 (2.10-2.40)	1.67±0.12 (1.50-1.90)
18	4.32±0.13 (4.10-4.50)	3.58±0.09 (3.40-3.70)	3.15±0.08 (3.00-3.30)	2.67±0.14 (2.50-2.90)	2.21±0.11 (2.10-2.40)	1.64±0.13 (1.50-1.80)
19	4.85±0.23 (4.50-5.30)	3.96±0.23 (3.60-4.30)	3.35±0.16 (3.10-3.60)	2.85±0.18 (2.60-3.20)	2.35±0.17 (2.20-2.70)	1.87±0.28 (1.50-2.30)
20	5.21±0.19 (4.90-5.50)	4.51±0.24 (4.10-4.80)	3.68±0.10 (3.60-3.90)	2.82±0.33 (2.40-3.30)	2.45±0.19 (2.10-2.70)	2.15±0.21 (1.70-2.50)
21	5.33±0.21 (5.00-5.60)	4.72±0.13 (4.50-4.90)	3.74±0.17 (3.50-4.00)	2.77±0.13 (2.60-3.00)	2.46±0.12 (2.30-2.60)	2.18±0.09 (2.00-2.30)
22	5.35±0.15 (5.10-5.50)	4.70±0.12 (4.50-4.90)	3.78±0.17 (3.50-4.00)	2.75±0.17 (2.50-3.00)	2.45±0.14 (2.20-2.70)	2.15±0.14 (1.90-2.40)

23	5.25±0.25 (4.90-5.60)	4.69±0.17 (4.40-4.90)	3.72±0.18 (3.50-4.00)	2.69±0.20 (2.40-3.00)	2.41±0.15 (2.20-2.60)	2.09±0.18 (1.80-2.30)
24	5.39±0.23 (5.00-5.70)	4.71±0.19 (4.30-4.90)	3.75±0.11 (3.60-3.90)	2.69±0.13 (2.50-2.90)	2.40±0.13 (2.20-2.60)	2.16±0.11 (2.00-2.30)
25	5.33±0.19 (5.00-5.60)	4.75±0.14 (4.50-4.90)	3.61±0.22 (3.30-4.00)	2.80±0.23 (2.50-3.10)	2.41±0.15 (2.20-2.60)	2.03±0.16 (1.80-2.30)
26	5.44±0.10 (5.30-5.60)	4.66±0.13 (4.50-4.90)	3.36±0.21 (3.00-3.70)	2.64±0.23 (2.40-3.20)	2.33±0.13 (2.20-2.60)	1.68±0.18 (1.50-2.10)
27	5.36±0.16 (5.10-5.60)	4.73±0.13 (4.50-4.90)	3.21±0.15 (3.00-3.50)	2.73±0.22 (2.50-3.10)	2.38±0.16 (2.10-2.60)	1.69±0.12 (1.50-1.90)
28	5.39±0.07 (5.30-5.50)	4.65±0.11 (4.50-4.80)	3.40±0.17 (3.20-3.70)	2.65±0.12 (2.50-2.80)	2.34±0.11 (2.20-2.50)	1.75±0.19 (1.50-2.00)
29	5.51±0.26 (5.10-5.90)	4.81±0.25 (4.50-5.20)	3.32±0.20 (3.00-3.60)	2.63±0.11 (2.50-2.80)	2.27±0.08 (2.20-2.40)	1.94±0.21 (1.60-2.30)
30	5.54±0.11 (5.30-5.70)	4.76±0.23 (4.50-5.20)	3.78±0.33 (3.50-4.50)	2.72±0.23 (2.40-3.20)	2.32±0.21 (2.10-2.80)	1.92±0.24 (1.60-2.40)
31	5.46±0.22 (5.10-5.80)	4.62±0.44 (4.10-5.60)	3.68±0.23 (3.40-4.20)	2.65±0.18 (2.40-2.90)	2.47±0.19 (2.20-2.70)	2.04±0.16 (1.80-2.30)
32	5.54±0.17 (5.30-5.80)	4.62±0.14 (4.40-4.90)	3.60±0.08 (3.50-3.70)	2.59±0.10 (2.40-2.70)	2.39±0.17 (2.10-2.60)	2.04±0.20 (1.80-2.30)
33	5.49±0.18 (5.20-5.70)	4.58±0.11 (4.40-4.80)	3.60±0.14 (3.40-3.90)	2.61±0.15 (2.40-2.90)	2.39±0.19 (2.20-2.70)	2.15±0.18 (1.90-2.40)
34	5.52±0.13 (5.30-5.70)	4.57±0.13 (4.40-4.80)	3.50±0.12 (3.30-3.70)	2.58±0.12 (2.40-2.80)	2.33±0.18 (2.10-2.70)	2.04±0.18 (1.80-2.40)
35	5.44±0.13 (5.20-5.60)	4.66±0.16 (4.40-4.90)	3.60±0.14 (3.40-3.80)	2.58±0.10 (2.40-2.70)	2.36±0.11 (2.20-2.50)	2.11±0.12 (1.90-2.30)
36	5.51±0.14 (5.30-5.70)	4.61±0.19 (4.30-4.80)	3.63±0.22 (3.30-4.00)	2.72±0.15 (2.50-2.90)	2.54±0.21 (2.20-2.80)	2.19±0.21 (1.80-2.50)
37	5.42±0.25 (5.00-5.70)	4.56±0.19 (4.20-4.80)	3.69±0.33 (3.30-4.50)	2.60±0.15 (2.40-2.90)	2.41±0.18 (2.10-2.70)	2.11±0.20 (1.80-2.50)
38	5.68±0.18 (5.50-6.00)	4.71±0.23 (4.20-5.00)	3.78±0.46 (3.20-4.60)	2.66±0.17 (2.40-2.90)	2.43±0.20 (2.10-2.70)	2.22±0.27 (1.80-2.60)
39	5.67±0.26 (5.40-6.10)	4.77±0.24 (4.40-5.20)	3.76±0.23 (3.20-4.00)	2.66±0.13 (2.50-2.90)	2.44±0.19 (2.20-2.70)	2.27±0.19 (2.00-2.50)
40	5.54±0.14 (5.30-5.80)	4.66±0.20 (4.30-4.90)	3.49±0.31 (2.80-3.90)	2.91±0.47 (2.40-3.50)	2.41±0.18 (2.10-2.60)	2.16±0.14 (1.90-2.30)
41	5.65±0.14 (5.50-5.90)	4.50±0.19 (4.20-4.80)	3.55±0.20 (3.20-3.80)	3.18±0.16 (2.80-3.30)	2.61±0.12 (2.40-2.80)	2.32±0.12 (2.10-2.50)
42	5.53±0.12 (5.30-5.70)	4.57±0.20 (4.20-4.80)	3.70±0.16 (3.40-3.90)	3.32±0.10 (3.20-3.50)	2.63±0.09 (2.50-2.80)	2.37±0.12 (2.20-2.50)
43	5.58±0.10 (5.40-5.70)	4.60±0.12 (4.40-4.70)	3.53±0.22 (3.20-3.80)	3.07±0.23 (2.70-3.40)	2.59±0.18 (2.40-2.90)	2.29±0.26 (1.90-2.70)
44	5.47±0.29 (5.00-6.00)	4.49±0.22 (4.20-5.00)	3.65±0.24 (3.20-4.20)	2.97±0.18 (2.70-3.30)	2.51±0.12 (2.30-2.70)	2.21±0.17 (2.00-2.60)
45	5.54±0.13 (5.40-5.80)	4.65±0.13 (4.50-4.90)	3.68±0.18 (3.50-4.00)	3.16±0.10 (3.00-3.30)	2.59±0.07 (2.50-2.70)	2.32±0.10 (2.20-2.50)
46	5.73±0.26 (5.40-6.20)	4.76±0.21 (4.50-5.00)	3.74±0.22 (3.40-4.10)	3.31±0.16 (3.10-3.60)	2.59±0.11 (2.50-2.80)	2.26±0.19 (2.00-2.60)
47	5.72±0.15 (5.50-5.90)	4.78±0.14 (4.50-4.90)	3.73±0.14 (3.50-3.90)	3.12±0.20 (2.80-3.40)	2.58±0.12 (2.40-2.80)	2.32±0.15 (2.10-2.60)
48	5.71±0.17 (5.50-6.00)	4.81±0.14 (4.60-5.00)	3.77±0.17 (3.50-4.00)	2.85±0.25 (2.50-3.20)	2.45±0.16 (2.20-2.70)	2.20±0.14 (2.00-2.50)

49	5.72±0.19 (5.50-6.00)	4.80±0.18 (4.60-5.10)	3.73±0.15 (3.50-3.90)	2.78±0.15 (2.50-3.00)	2.50±0.18 (2.20-2.70)	2.18±0.20 (1.90-2.50)
50	5.82±0.18 (5.50-6.10)	4.88±0.11 (4.70-5.10)	3.70±0.16 (3.50-4.00)	2.90±0.16 (2.70-3.20)	2.64±0.14 (2.50-2.90)	2.36±0.18 (2.20-2.70)
51	5.73±0.19 (5.50-6.00)	4.78±0.16 (4.60-5.00)	3.51±0.19 (3.30-3.80)	2.75±0.08 (2.60-2.90)	2.47±0.18 (2.00-2.70)	2.19±0.21 (1.80-2.60)
52	5.70±0.23 (5.40-6.10)	4.76±0.18 (4.50-5.10)	3.60±0.17 (3.40-3.90)	2.78±0.13 (2.60-3.00)	2.58±0.11 (2.40-2.70)	2.29±0.10 (2.20-2.50)
53	5.65±0.14 (5.40-5.80)	4.74±0.13 (4.50-4.90)	3.67±0.13 (3.40-3.80)	2.75±0.08 (2.60-2.90)	2.55±0.11 (2.40-2.80)	2.21±0.07 (2.10-2.30)
54	5.65±0.17 (5.40-6.00)	4.67±0.12 (4.50-4.80)	3.49±0.19 (3.20-3.90)	2.78±0.12 (2.60-3.00)	2.59±0.17 (2.30-2.80)	2.26±0.17 (1.90-2.50)
55	5.78±0.15 (5.60-6.00)	4.71±0.20 (4.40-5.00)	3.63±0.30 (3.20-4.00)	2.86±0.13 (2.70-3.10)	2.63±0.14 (2.50-2.90)	2.41±0.19 (2.20-2.70)
56	5.69±0.14 (5.50-5.90)	4.60±0.19 (4.20-4.80)	3.48±0.22 (3.20-4.00)	2.79±0.10 (2.60-2.90)	2.55±0.07 (2.50-2.70)	2.26±0.13 (2.10-2.50)
57	5.75±0.11 (5.60-5.90)	4.60±0.17 (4.30-4.90)	3.53±0.16 (3.30-3.80)	2.71±0.12 (2.50-2.90)	2.50±0.18 (2.20-2.70)	2.22±0.28 (1.80-2.50)
58	5.78±0.14 (5.50-5.90)	4.67±0.18 (4.50-5.00)	3.62±0.22 (3.30-3.90)	2.80±0.21 (2.40-3.10)	2.41±0.07 (2.30-2.50)	2.08±0.11 (1.90-2.30)
59	5.89±0.12 (5.70-6.10)	4.70±0.12 (4.50-4.90)	3.65±0.14 (3.40-3.90)	2.93±0.17 (2.60-3.10)	2.73±0.17 (2.40-2.90)	2.53±0.23 (2.00-2.80)
60	5.71±0.11 (5.50-5.90)	4.59±0.17 (4.30-4.80)	3.63±0.16 (3.40-3.90)	2.72±0.15 (2.50-3.00)	2.45±0.14 (2.20-2.70)	2.12±0.18 (1.80-2.30)
61	5.77±0.16 (5.50-6.00)	4.67±0.27 (4.00-5.00)	3.62±0.13 (3.40-3.80)	2.71±0.13 (2.50-2.90)	2.56±0.16 (2.30-2.80)	2.30±0.29 (1.80-2.70)
62	5.81±0.17 (5.60-6.10)	4.65±0.18 (4.40-4.90)	3.52±0.22 (3.00-3.70)	2.70±0.12 (2.50-2.90)	2.46±0.22 (2.00-2.70)	2.23±0.28 (1.70-2.60)
63	5.70±0.09 (5.60-5.90)	4.63±0.11 (4.50-4.80)	3.55±0.21 (3.30-3.90)	2.87±0.13 (2.70-3.10)	2.69±0.14 (2.50-2.90)	2.44±0.16 (2.20-2.70)
64	5.84±0.13 (5.70-6.10)	4.70±0.14 (4.50-4.90)	3.46±0.10 (3.30-3.60)	2.83±0.16 (2.50-3.10)	2.65±0.20 (2.20-2.90)	2.31±0.22 (1.90-2.70)
65	5.76±0.14 (5.60-6.00)	4.58±0.09 (4.40-4.70)	3.66±0.13 (3.50-3.90)	2.86±0.15 (2.60-3.10)	2.65±0.14 (2.50-2.90)	2.35±0.18 (2.10-2.70)
66	5.82±0.09 (5.70-6.00)	4.56±0.08 (4.40-4.70)	3.57±0.18 (3.30-3.80)	2.69±0.15 (2.50-3.00)	2.39±0.14 (2.20-2.60)	2.06±0.20 (1.80-2.40)
67	5.79±0.10 (5.60-5.90)	4.69±0.18 (4.50-5.00)	3.65±0.14 (3.50-3.90)	2.76±0.20 (2.50-3.10)	2.40±0.14 (2.20-2.60)	2.02±0.14 (1.80-2.20)
68	5.81±0.16 (5.60-6.00)	4.73±0.21 (4.50-5.00)	3.65±0.13 (3.50-3.90)	2.79±0.14 (2.60-3.00)	2.53±0.18 (2.30-2.90)	2.26±0.22 (1.90-2.60)
69	5.71±0.12 (5.50-5.90)	4.78±0.10 (4.60-4.90)	3.66±0.18 (3.40-3.90)	2.78±0.18 (2.50-3.10)	2.50±0.20 (2.10-2.80)	2.20±0.19 (1.80-2.50)
70	5.65±0.19 (5.30-5.90)	4.57±0.12 (4.40-4.80)	3.73±0.13 (3.50-3.90)	2.78±0.13 (2.60-3.00)	2.44±0.12 (2.20-2.60)	2.08±0.15 (1.80-2.30)
71	5.60±0.22 (5.30-6.00)	4.74±0.13 (4.50-4.90)	3.70±0.13 (3.50-3.90)	2.73±0.09 (2.60-2.90)	2.49±0.12 (2.30-2.70)	2.15±0.16 (1.90-2.50)
72	5.60±0.35 (5.00-6.20)	4.68±0.13 (4.50-4.90)	3.63±0.13 (3.40-3.80)	2.75±0.18 (2.50-3.00)	2.47±0.21 (2.20-2.80)	2.20±0.30 (1.80-2.60)
73	5.59±0.26 (5.10-6.00)	4.71±0.20 (4.50-5.00)	3.63±0.19 (3.20-3.90)	2.83±0.12 (2.60-3.00)	2.58±0.15 (2.30-2.90)	2.35±0.22 (2.00-2.80)
74	5.50±0.13 (5.30-5.70)	4.77±0.19 (4.50-5.00)	3.62±0.23 (3.30-4.00)	2.83±0.15 (2.60-3.10)	2.59±0.21 (2.20-2.90)	2.27±0.32 (1.70-2.70)

75	5.49±0.23 (5.20-6.00)	4.58±0.10 (4.50-4.80)	3.65±0.13 (3.50-3.90)	2.83±0.13 (2.60-3.00)	2.58±0.21 (2.30-2.90)	2.26±0.22 (1.90-2.60)
76	5.56±0.16 (5.30-5.80)	4.66±0.16 (4.50-4.90)	3.59±0.10 (3.40-3.70)	2.80±0.12 (2.60-3.00)	2.40±0.09 (2.20-2.50)	2.12±0.12 (1.90-2.30)
77	5.48±0.25 (5.20-5.90)	4.68±0.12 (4.50-4.90)	3.60±0.11 (3.50-3.80)	2.74±0.12 (2.60-3.00)	2.46±0.20 (2.20-2.90)	2.17±0.25 (1.90-2.70)
78	5.65±0.26 (5.40-6.00)	4.64±0.10 (4.50-4.80)	3.63±0.13 (3.40-3.80)	2.75±0.11 (2.60-2.90)	2.52±0.19 (2.20-2.70)	2.21±0.23 (1.80-2.50)
79	5.74±0.21 (5.50-6.20)	4.59±0.21 (4.30-5.00)	3.47±0.17 (3.20-3.70)	2.86±0.22 (2.50-3.20)	2.57±0.24 (2.10-2.90)	2.32±0.32 (1.70-2.70)
80	5.83±0.22 (5.50-6.20)	4.71±0.43 (4.30-5.50)	3.56±0.26 (3.20-4.00)	2.79±0.09 (2.70-2.90)	2.53±0.12 (2.40-2.70)	2.25±0.20 (2.00-2.50)
Total	5.21±0.76 (3.40-6.20)	4.35±0.61 (2.40-5.60)	3.43±0.41 (1.70-4.60)	2.75±0.25 (1.60-3.60)	2.43±0.23 (1.40-2.90)	2.09±0.31 (1.20-2.90)
p	< 0.001	< 0.001	< 0.001	< 0.001	< 0.001	< 0.001

Data are presented as mean ± standard deviation (observed minimum–maximum). The minimum–maximum values represent the observed ranges in the study sample and should not be interpreted as formal clinical reference intervals. SV1: diameter of the splenic vein at the proximal level, SV2: diameter of the splenic vein at the middle level, SV3: diameter of the splenic vein at the distal level, SA1: diameter of the splenic artery at the proximal level, SA2: diameter of the splenic artery at the middle level, SA3: diameter of the splenic artery at the distal level, $p < 0.05$ statistically significant.

Supplemental Digital Table 2: Ratios of the parameters to L1TD in all ages.

Age	SV1 / L1TD	SV2 / L1TD	SV3 / L1TD	SA1 / L1TD	SA2 / L1TD	SA3 / L1TD
1	0.17±0.01 (0.15-0.20)	0.14±0.02 (0.11-0.17)	0.11±0.02 (0.09-0.16)	0.09±0.02 (0.07-0.13)	0.08±0.02 (0.06-0.11)	0.07±0.03 (0.05-0.13)
2	0.16±0.02 (0.13-0.18)	0.13±0.01 (0.11-0.16)	0.11±0.01 (0.09-0.13)	0.10±0.01 (0.08-0.12)	0.08±0.01 (0.07-0.10)	0.07±0.01 (0.05-0.09)
3	0.15±0.01 (0.13-0.17)	0.13±0.01 (0.11-0.14)	0.10±0.01 (0.07-0.12)	0.10±0.01 (0.08-0.11)	0.08±0.01 (0.07-0.09)	0.06±0.01 (0.05-0.07)
4	0.14±0.01 (0.12-0.16)	0.12±0.01 (0.11-0.13)	0.10±0.01 (0.09-0.12)	0.10±0.01 (0.07-0.11)	0.08±0.01 (0.05-0.09)	0.07±0.01 (0.04-0.09)
5	0.13±0.01 (0.11-0.16)	0.10±0.01 (0.08-0.12)	0.09±0.01 (0.07-0.10)	0.09±0.01 (0.08-0.11)	0.07±0.01 (0.06-0.09)	0.06±0.01 (0.05-0.07)
6	0.12±0.01 (0.11-0.13)	0.10±0.01 (0.09-0.11)	0.08±0.01 (0.07-0.09)	0.08±0.00 (0.08-0.09)	0.07±0.00 (0.07-0.07)	0.06±0.01 (0.05-0.07)
7	0.11±0.01 (0.10-0.12)	0.10±0.01 (0.09-0.11)	0.08±0.01 (0.05-0.09)	0.08±0.01 (0.07-0.09)	0.07±0.01 (0.06-0.08)	0.06±0.01 (0.04-0.07)
8	0.11±0.01 (0.09-0.12)	0.09±0.01 (0.08-0.10)	0.08±0.01 (0.07-0.10)	0.08±0.01 (0.07-0.09)	0.07±0.01 (0.06-0.08)	0.06±0.01 (0.05-0.08)
9	0.10±0.01 (0.09-0.12)	0.09±0.01 (0.07-0.10)	0.08±0.01 (0.07-0.09)	0.08±0.01 (0.07-0.09)	0.07±0.00 (0.06-0.08)	0.07±0.00 (0.06-0.07)
10	0.10±0.01 (0.10-0.12)	0.08±0.01 (0.08-0.10)	0.08±0.01 (0.07-0.09)	0.07±0.01 (0.06-0.08)	0.06±0.01 (0.06-0.08)	0.05±0.01 (0.04-0.06)
11	0.09±0.01 (0.08-0.11)	0.08±0.01 (0.07-0.10)	0.07±0.01 (0.06-0.09)	0.07±0.01 (0.06-0.08)	0.06±0.01 (0.05-0.07)	0.05±0.01 (0.04-0.06)
12	0.10±0.01 (0.09-0.11)	0.08±0.01 (0.08-0.10)	0.07±0.01 (0.06-0.08)	0.06±0.01 (0.05-0.07)	0.05±0.00 (0.05-0.06)	0.04±0.00 (0.03-0.05)
13	0.09±0.01 (0.08-0.10)	0.08±0.01 (0.07-0.09)	0.06±0.01 (0.06-0.07)	0.06±0.01 (0.05-0.07)	0.05±0.00 (0.04-0.06)	0.04±0.01 (0.03-0.05)
14	0.09±0.01 (0.08-0.10)	0.08±0.01 (0.07-0.09)	0.07±0.00 (0.06-0.08)	0.07±0.01 (0.06-0.07)	0.06±0.01 (0.05-0.06)	0.05±0.01 (0.04-0.05)
15	0.10±0.01 (0.09-0.11)	0.08±0.01 (0.07-0.09)	0.07±0.01 (0.07-0.08)	0.06±0.00 (0.05-0.06)	0.05±0.00 (0.05-0.06)	0.04±0.01 (0.03-0.05)
16	0.10±0.01 (0.09-0.12)	0.08±0.01 (0.07-0.09)	0.07±0.01 (0.06-0.08)	0.06±0.01 (0.05-0.07)	0.05±0.01 (0.04-0.06)	0.04±0.00 (0.03-0.04)
17	0.09±0.01 (0.08-0.11)	0.08±0.01 (0.07-0.09)	0.07±0.01 (0.06-0.08)	0.06±0.01 (0.05-0.07)	0.05±0.00 (0.04-0.05)	0.04±0.00 (0.03-0.04)
18	0.10±0.00 (0.09-0.10)	0.08±0.01 (0.07-0.09)	0.07±0.00 (0.06-0.08)	0.06±0.01 (0.05-0.07)	0.05±0.00 (0.04-0.06)	0.04±0.00 (0.03-0.04)
19	0.11±0.01 (0.09-0.12)	0.09±0.01 (0.07-0.10)	0.07±0.01 (0.06-0.08)	0.06±0.01 (0.05-0.08)	0.05±0.01 (0.04-0.06)	0.04±0.01 (0.03-0.05)
20	0.12±0.01 (0.10-0.13)	0.10±0.01 (0.08-0.12)	0.08±0.01 (0.07-0.10)	0.06±0.01 (0.05-0.08)	0.05±0.01 (0.04-0.06)	0.05±0.01 (0.03-0.06)
21	0.12±0.02 (0.09-0.15)	0.10±0.01 (0.08-0.13)	0.08±0.01 (0.07-0.11)	0.06±0.01 (0.05-0.08)	0.05±0.01 (0.05-0.07)	0.05±0.01 (0.04-0.06)
22	0.12±0.01 (0.10-0.13)	0.10±0.01 (0.09-0.11)	0.08±0.01 (0.07-0.09)	0.06±0.01 (0.05-0.07)	0.05±0.00 (0.05-0.06)	0.05±0.00 (0.04-0.05)
23	0.12±0.01 (0.10-0.14)	0.10±0.01 (0.09-0.12)	0.08±0.01 (0.07-0.09)	0.06±0.01 (0.05-0.07)	0.05±0.01 (0.05-0.06)	0.05±0.01 (0.04-0.05)
24	0.12±0.01 (0.10-0.14)	0.11±0.01 (0.09-0.12)	0.08±0.01 (0.07-0.10)	0.06±0.01 (0.05-0.07)	0.05±0.01 (0.04-0.06)	0.05±0.01 (0.04-0.06)

25	0.12±0.01 (0.10-0.14)	0.11±0.01 (0.09-0.12)	0.08±0.01 (0.07-0.09)	0.06±0.01 (0.05-0.07)	0.05±0.00 (0.04-0.06)	0.04±0.00 (0.04-0.05)
26	0.12±0.01 (0.11-0.13)	0.11±0.01 (0.10-0.11)	0.08±0.00 (0.07-0.08)	0.06±0.01 (0.05-0.07)	0.05±0.01 (0.05-0.06)	0.04±0.00 (0.03-0.05)
27	0.12±0.01 (0.11-0.14)	0.10±0.01 (0.10-0.12)	0.07±0.01 (0.06-0.08)	0.06±0.01 (0.05-0.08)	0.05±0.01 (0.04-0.06)	0.04±0.00 (0.03-0.04)
28	0.12±0.01 (0.10-0.13)	0.10±0.01 (0.08-0.11)	0.07±0.00 (0.07-0.08)	0.06±0.01 (0.05-0.07)	0.05±0.01 (0.04-0.06)	0.04±0.01 (0.03-0.05)
29	0.11±0.01 (0.10-0.13)	0.10±0.01 (0.08-0.11)	0.07±0.01 (0.06-0.08)	0.05±0.01 (0.05-0.06)	0.05±0.00 (0.04-0.05)	0.04±0.01 (0.03-0.05)
30	0.12±0.01 (0.11-0.15)	0.10±0.01 (0.09-0.14)	0.08±0.01 (0.07-0.12)	0.06±0.01 (0.05-0.07)	0.05±0.01 (0.04-0.07)	0.04±0.01 (0.03-0.06)
31	0.12±0.01 (0.11-0.13)	0.10±0.01 (0.09-0.11)	0.08±0.01 (0.07-0.09)	0.06±0.01 (0.05-0.07)	0.05±0.01 (0.05-0.06)	0.04±0.00 (0.04-0.05)
32	0.12±0.01 (0.10-0.13)	0.10±0.01 (0.09-0.11)	0.08±0.00 (0.07-0.08)	0.06±0.00 (0.05-0.06)	0.05±0.00 (0.05-0.06)	0.04±0.00 (0.04-0.05)
33	0.12±0.01 (0.10-0.14)	0.10±0.01 (0.08-0.11)	0.08±0.01 (0.07-0.09)	0.06±0.01 (0.05-0.07)	0.05±0.01 (0.04-0.06)	0.05±0.01 (0.04-0.06)
34	0.12±0.01 (0.10-0.14)	0.10±0.01 (0.08-0.11)	0.08±0.01 (0.07-0.09)	0.06±0.01 (0.05-0.06)	0.05±0.00 (0.04-0.06)	0.04±0.00 (0.04-0.05)
35	0.12±0.01 (0.11-0.13)	0.10±0.01 (0.09-0.11)	0.08±0.01 (0.07-0.09)	0.06±0.00 (0.05-0.06)	0.05±0.00 (0.04-0.06)	0.05±0.00 (0.04-0.05)
36	0.12±0.01 (0.12-0.13)	0.10±0.01 (0.10-0.11)	0.08±0.01 (0.07-0.09)	0.06±0.00 (0.06-0.07)	0.06±0.01 (0.05-0.07)	0.05±0.01 (0.04-0.06)
37	0.12±0.01 (0.11-0.13)	0.10±0.01 (0.09-0.11)	0.08±0.01 (0.07-0.09)	0.06±0.01 (0.05-0.07)	0.05±0.01 (0.04-0.06)	0.05±0.01 (0.04-0.06)
38	0.12±0.01 (0.11-0.13)	0.10±0.01 (0.09-0.11)	0.08±0.01 (0.06-0.09)	0.06±0.00 (0.05-0.06)	0.05±0.01 (0.04-0.06)	0.05±0.01 (0.04-0.05)
39	0.12±0.01 (0.10-0.14)	0.10±0.01 (0.09-0.12)	0.08±0.01 (0.07-0.09)	0.06±0.01 (0.05-0.06)	0.05±0.00 (0.05-0.06)	0.05±0.00 (0.04-0.06)
40	0.11±0.01 (0.10-0.12)	0.09±0.01 (0.08-0.11)	0.07±0.01 (0.06-0.08)	0.06±0.01 (0.04-0.07)	0.05±0.00 (0.04-0.05)	0.04±0.00 (0.04-0.05)
41	0.12±0.01 (0.10-0.14)	0.10±0.01 (0.09-0.11)	0.08±0.01 (0.07-0.09)	0.07±0.01 (0.06-0.08)	0.06±0.00 (0.05-0.06)	0.05±0.00 (0.05-0.06)
42	0.11±0.01 (0.10-0.13)	0.09±0.01 (0.08-0.11)	0.08±0.01 (0.07-0.09)	0.07±0.01 (0.06-0.08)	0.05±0.00 (0.05-0.07)	0.05±0.00 (0.04-0.06)
43	0.12±0.00 (0.12-0.12)	0.10±0.01 (0.09-0.11)	0.08±0.01 (0.07-0.09)	0.07±0.01 (0.06-0.08)	0.06±0.01 (0.05-0.06)	0.05±0.01 (0.04-0.06)
44	0.11±0.01 (0.10-0.14)	0.09±0.01 (0.08-0.11)	0.08±0.01 (0.06-0.10)	0.06±0.00 (0.05-0.07)	0.05±0.00 (0.05-0.06)	0.05±0.00 (0.04-0.05)
45	0.12±0.01 (0.10-0.15)	0.10±0.01 (0.08-0.12)	0.08±0.01 (0.07-0.10)	0.07±0.01 (0.06-0.08)	0.05±0.01 (0.05-0.07)	0.05±0.00 (0.04-0.06)
46	0.12±0.01 (0.10-0.14)	0.10±0.01 (0.09-0.12)	0.08±0.01 (0.06-0.10)	0.07±0.01 (0.06-0.08)	0.05±0.01 (0.05-0.07)	0.05±0.01 (0.04-0.06)
47	0.12±0.01 (0.11-0.13)	0.10±0.01 (0.09-0.11)	0.08±0.00 (0.07-0.08)	0.06±0.00 (0.06-0.07)	0.05±0.00 (0.05-0.06)	0.05±0.00 (0.04-0.05)
48	0.12±0.01 (0.11-0.13)	0.10±0.01 (0.09-0.11)	0.08±0.01 (0.07-0.09)	0.06±0.00 (0.05-0.07)	0.05±0.00 (0.05-0.06)	0.05±0.00 (0.04-0.05)
49	0.12±0.01 (0.10-0.13)	0.10±0.01 (0.08-0.11)	0.08±0.01 (0.07-0.08)	0.06±0.01 (0.04-0.06)	0.05±0.01 (0.04-0.06)	0.04±0.01 (0.03-0.05)
50	0.12±0.01 (0.11-0.13)	0.10±0.01 (0.09-0.11)	0.07±0.00 (0.07-0.08)	0.06±0.00 (0.05-0.06)	0.05±0.00 (0.05-0.06)	0.05±0.00 (0.04-0.05)

51	0.12±0.01 (0.10-0.13)	0.10±0.01 (0.09-0.11)	0.07±0.01 (0.06-0.08)	0.06±0.00 (0.05-0.06)	0.05±0.00 (0.04-0.06)	0.05±0.00 (0.04-0.05)
52	0.12±0.02 (0.09-0.14)	0.10±0.01 (0.08-0.12)	0.07±0.01 (0.06-0.09)	0.06±0.01 (0.05-0.07)	0.05±0.01 (0.04-0.06)	0.05±0.01 (0.04-0.06)
53	0.11±0.01 (0.09-0.13)	0.10±0.01 (0.08-0.11)	0.07±0.01 (0.05-0.09)	0.06±0.01 (0.04-0.06)	0.05±0.01 (0.04-0.06)	0.04±0.00 (0.03-0.05)
54	0.12±0.01 (0.10-0.13)	0.10±0.01 (0.08-0.11)	0.07±0.01 (0.06-0.08)	0.06±0.00 (0.05-0.07)	0.05±0.00 (0.05-0.06)	0.05±0.00 (0.04-0.06)
55	0.12±0.01 (0.10-0.14)	0.10±0.01 (0.08-0.12)	0.07±0.01 (0.06-0.09)	0.06±0.00 (0.05-0.07)	0.05±0.00 (0.05-0.06)	0.05±0.00 (0.04-0.05)
56	0.12±0.01 (0.10-0.14)	0.09±0.01 (0.08-0.11)	0.07±0.01 (0.05-0.09)	0.06±0.01 (0.05-0.07)	0.05±0.01 (0.04-0.06)	0.05±0.01 (0.04-0.06)
57	0.12±0.01 (0.11-0.13)	0.09±0.01 (0.08-0.11)	0.07±0.00 (0.07-0.08)	0.06±0.00 (0.05-0.06)	0.05±0.00 (0.04-0.06)	0.05±0.01 (0.04-0.05)
58	0.12±0.01 (0.10-0.13)	0.09±0.01 (0.08-0.11)	0.07±0.01 (0.06-0.08)	0.06±0.01 (0.05-0.07)	0.05±0.00 (0.04-0.05)	0.04±0.00 (0.04-0.05)
59	0.12±0.01 (0.10-0.14)	0.10±0.01 (0.08-0.11)	0.07±0.01 (0.06-0.08)	0.06±0.01 (0.05-0.07)	0.06±0.01 (0.05-0.06)	0.05±0.01 (0.04-0.06)
60	0.11±0.01 (0.10-0.12)	0.09±0.01 (0.08-0.10)	0.07±0.01 (0.06-0.08)	0.05±0.00 (0.05-0.06)	0.05±0.00 (0.04-0.05)	0.04±0.00 (0.04-0.05)
61	0.11±0.01 (0.10-0.12)	0.09±0.01 (0.08-0.11)	0.07±0.01 (0.06-0.08)	0.05±0.00 (0.05-0.06)	0.05±0.00 (0.05-0.06)	0.05±0.01 (0.04-0.05)
62	0.11±0.01 (0.10-0.13)	0.09±0.01 (0.08-0.10)	0.07±0.01 (0.06-0.08)	0.05±0.01 (0.04-0.06)	0.05±0.01 (0.04-0.06)	0.04±0.01 (0.03-0.06)
63	0.11±0.01 (0.10-0.13)	0.09±0.01 (0.08-0.10)	0.07±0.01 (0.06-0.08)	0.06±0.01 (0.05-0.07)	0.05±0.01 (0.04-0.06)	0.05±0.01 (0.04-0.06)
64	0.12±0.01 (0.11-0.14)	0.10±0.01 (0.09-0.11)	0.07±0.01 (0.06-0.08)	0.06±0.01 (0.05-0.07)	0.05±0.01 (0.05-0.07)	0.05±0.01 (0.04-0.06)
65	0.12±0.01 (0.10-0.13)	0.09±0.01 (0.08-0.11)	0.07±0.01 (0.06-0.08)	0.06±0.01 (0.05-0.07)	0.05±0.01 (0.05-0.07)	0.05±0.13 (0.04-0.06)
66	0.12±0.01 (0.10-0.13)	0.09±0.01 (0.08-0.11)	0.07±0.01 (0.06-0.09)	0.05±0.01 (0.05-0.07)	0.05±0.01 (0.04-0.06)	0.04±0.01 (0.03-0.05)
67	0.12±0.01 (0.10-0.14)	0.10±0.01 (0.08-0.12)	0.08±0.01 (0.06-0.09)	0.06±0.00 (0.05-0.06)	0.05±0.00 (0.04-0.06)	0.04±0.00 (0.04-0.05)
68	0.12±0.01 (0.11-0.14)	0.10±0.01 (0.09-0.12)	0.08±0.01 (0.07-0.09)	0.06±0.00 (0.05-0.06)	0.05±0.00 (0.05-0.06)	0.05±0.01 (0.04-0.06)
69	0.12±0.01 (0.10-0.14)	0.10±0.01 (0.08-0.12)	0.07±0.00 (0.07-0.08)	0.06±0.01 (0.05-0.07)	0.05±0.01 (0.04-0.06)	0.05±0.01 (0.04-0.05)
70	0.12±0.00 (0.10-0.12)	0.09±0.00 (0.09-0.10)	0.08±0.00 (0.07-0.08)	0.06±0.00 (0.05-0.06)	0.05±0.00 (0.05-0.05)	0.04±0.00 (0.04-0.05)
71	0.11±0.01 (0.10-0.13)	0.10±0.01 (0.09-0.10)	0.08±0.00 (0.07-0.08)	0.06±0.00 (0.05-0.06)	0.05±0.00 (0.04-0.06)	0.04±0.00 (0.04-0.05)
72	0.11±0.02 (0.10-0.15)	0.10±0.01 (0.08-0.11)	0.07±0.01 (0.06-0.09)	0.06±0.01 (0.05-0.07)	0.05±0.01 (0.04-0.06)	0.05±0.01 (0.03-0.06)
73	0.11±0.01 (0.09-0.14)	0.09±0.01 (0.08-0.11)	0.07±0.01 (0.06-0.08)	0.06±0.01 (0.05-0.07)	0.05±0.01 (0.04-0.07)	0.05±0.01 (0.04-0.06)
74	0.11±0.01 (0.10-0.12)	0.09±0.01 (0.08-0.10)	0.07±0.01 (0.06-0.08)	0.06±0.00 (0.05-0.06)	0.05±0.00 (0.04-0.06)	0.04±0.01 (0.03-0.05)
75	0.10±0.01 (0.09-0.12)	0.09±0.01 (0.08-0.10)	0.07±0.00 (0.06-0.08)	0.05±0.00 (0.05-0.06)	0.05±0.00 (0.04-0.06)	0.04±0.00 (0.03-0.05)
76	0.11±0.01 (0.10-0.12)	0.09±0.01 (0.08-0.10)	0.07±0.01 (0.06-0.08)	0.06±0.01 (0.05-0.07)	0.05±0.00 (0.04-0.06)	0.04±0.00 (0.04-0.05)

77	0.11±0.01 (0.10-0.12)	0.09±0.01 (0.08-0.10)	0.07±0.00 (0.06-0.08)	0.05±0.00 (0.05-0.06)	0.05±0.01 (0.04-0.06)	0.04±0.01 (0.04-0.06)
78	0.11±0.01 (0.09-0.15)	0.09±0.01 (0.08-0.11)	0.07±0.01 (0.06-0.09)	0.05±0.01 (0.04-0.07)	0.05±0.01 (0.04-0.06)	0.04±0.01 (0.03-0.06)
79	0.12±0.01 (0.11-0.13)	0.09±0.01 (0.08-0.11)	0.07±0.00 (0.06-0.08)	0.06±0.01 (0.05-0.07)	0.05±0.01 (0.04-0.07)	0.05±0.01 (0.04-0.06)
80	0.12±0.02 (0.10-0.14)	0.09±0.02 (0.08-0.13)	0.07±0.01 (0.06-0.09)	0.06±0.01 (0.05-0.07)	0.05±0.01 (0.04-0.07)	0.05±0.01 (0.04-0.06)
Total	0.12±0.02 (0.08-0.20)	0.10±0.01 (0.07-0.17)	0.08±0.01 (0.05-0.16)	0.06±0.01 (0.04-0.13)	0.05±0.01 (0.04-0.11)	0.05±0.02 (0.03-0.13)
p	< 0.001	< 0.001	< 0.001	< 0.001	< 0.001	< 0.001

Data are presented as mean ± standard deviation (observed minimum–maximum). The minimum–maximum values represent the observed ranges in the study sample and should not be interpreted as formal clinical reference intervals. SV1: diameter of the splenic vein at the proximal level, SV2: diameter of the splenic vein at the middle level, SV3: diameter of the splenic vein at the distal level, SA1: diameter of the splenic artery at the proximal level, SA2: diameter of the splenic artery at the middle level, SA3: diameter of the splenic artery at the distal level, L1TD: transverse diameter of L1's body, $p < 0.05$ statistically significant.
